# Re-detectable positive SARS-CoV-2 RNA tests in patients who recovered from COVID-19 with intestinal infection

**DOI:** 10.1007/s13238-020-00778-8

**Published:** 2020-09-26

**Authors:** Wanyin Tao, Xiaofang Wang, Guorong Zhang, Meng Guo, Huan Ma, Dan Zhao, Yong Sun, Jun He, Lianxin Liu, Kaiguang Zhang, Yucai Wang, Jianping Weng, Xiaoling Ma, Tengchuan Jin, Shu Zhu

**Affiliations:** 1 The First Affiliated Hospital of USTC, Division of Life Sciences and Medicine, University of Science and Technology of China, Hefei, China; 2 Hefei National Laboratory for Physical Sciences at Microscale, the CAS Key Laboratory of Innate Immunity and Chronic Disease, School of Basic Medical Sciences, Division of Life Sciences and Medicine, University of Science and Technology of China, Hefei, China; 3 School of Data Science, University of Science and Technology of China, Hefei, China; 4 CAS Centre for Excellence in Cell and Molecular Biology, University of Science and Technology of China, Hefei, China; 5 Key Laboratory for Medical and Health of the 13th Five-Year Plan, Anhui Provincial Center for Disease Control and Prevention, Hefei, China

**Dear Editor,**

Severe acute respiratory syndrome coronavirus 2 (SARS-CoV-2), a novel coronavirus that causes Coronavirus Disease 2019 (COVID-19) (Yang and Wang, 2020), has spread to more than 200 countries and infected more than 9,000,000 people as of Jun 2020. Tens of thousands of patients with COVID-19 have recovered and been discharged from hospital. However, there are reports of recovered patients who subsequently tested positive for SARS-CoV-2 after discharge (re-detectable positive, RP) (An et al., 2020; Lan et al., 2020), and this has led to increasing focus on the mechanism(s) underlying RP.

Several studies of discharged patients have been performed to investigate the proportion of RP and the clinical symptoms of these patients (An et al., 2020; Lan et al., 2020). One study revealed that four patients had positive SARS-CoV-2 RT-PCR tests 5 to 13 days after discharge (Lan et al., 2020), while a retrospective study in Wuhan, China reported that 8/108 (7.4%) patients were RP (Cao et al., 2020). Moreover, 14.5% of convalescent patients (*n* = 38) were RP for SARS-CoV-2 RNA in RT-PCR tests of both anal and nasopharyngeal swabs (An et al., 2020). RP is usually observed in young patients who had mild or moderate COVID-19 symptoms on the first admission, and several significant characteristic features including early RNA-negative conversion, fewer comorbidities, and more frequent upper respiratory symptoms (An et al., 2020). No obvious clinical symptoms were reported on the second admission of these patients (An et al., 2020; Lan et al., 2020; Cao et al., 2020). In recent reports, close contacts of RP patients were tested negative for SARS-CoV-2 RNA (An et al., 2020; Lan et al., 2020). However, as the patients were usually in quarantine after discharge, the infectivity of the patients might be underestimated. Two patients who were RP continued to be positive for SARS-COV-2 RNA for more than 90 days (Cao et al., 2020). Careful consideration should therefore be given to the potential for patients who are RP to become chronic virus-carriers.

The cause of RP remains controversial, and consequently it is difficult to set standards for discharge and follow-up of patients. False negatives in qRT-PCR tests may partially explain the RP in some cases, because the lower limit of detection (LOD) of commercial RT-PCR kits is relatively high (An et al., 2020). The residual viremia could be another factor that leads to RP. Pathological examination of a ready-for-discharge patient who had COVID-19 identified viral particles in the pneumocytes. Several studies have reported the presence of SARS-CoV-2 RNA and viral particles in the gastrointestinal tracts of patients who had COVID-19, and that fecal samples remained viral RNA-positive after patients were respiratory-negative for SARS-CoV-2 and were discharged from hospital (Xu et al., 2020), thus highlighting the prolonged presence of SARS-CoV-2 in the gastrointestinal tract.

Here, we investigated whether the intestine might be a “reservoir” of SARS-CoV-2 and one of the potential causes of RP. Between January 21 and March 8, 2020, a total of 173 patients who had COVID-19 were discharged from hospitals in Hefei, China, including the First Affiliated Hospital of University of Science and Technology of China (USTC). The patients were enrolled in this study for analysis of clinical parameters, and were followed for at least one month. During the monitoring, 12 out of 173 patients were found to be RP (Table S1). All 12 of these patients had gastrointestinal symptoms, including nausea, diarrhea, anorexia, abdominal pain and belching (Table S2), and three out of those patients who were RP developed a gastrointestinal symptom as the onset symptom (Table S2). In contrast to the reported 2%–39% of patients with COVID-19 who develop gastrointestinal symptoms (Chen et al., 2020; Zhang et al., 2020; Zheng et al., 2020), patients who were RP in Hefei had a much higher incidence of gastrointestinal symptoms (*P*-value < 2.2 × 10^−16^ by Chi-squared contingency table test).

Patient G developed a fever and diarrhea over 3 days from January 28, 2020, was confirmed to have COVID-19 on February 4, and was then transferred to the intensive care unit (ICU) of the First Affiliated Hospital of USTC as a severe case. The patient experienced respiratory distress from February 4 to February 9. He was given glucocorticoid, immunoglobin, antibiotics, antiviral agents, tocilizumab (anti-IL6R), and probiotics, and supplied with oxygen. The respiratory distress symptoms significantly improved from February 11, and the patient was transferred out of the ICU on February 13. SARS-CoV-2 RNA tests on throat swab samples from the patient were negative on February 12 and 14, and he was discharged from hospital on February 20. After discharge, the patient remained in quarantine, and was tested for SARS-CoV-2 RNA every three days. A throat swab sample from the patient was again positive for SARS-CoV-2 RNA on March 5 (Fig. 1A). Unfortunately, fecal samples from Patient G were not collected.

**Figure 1:**
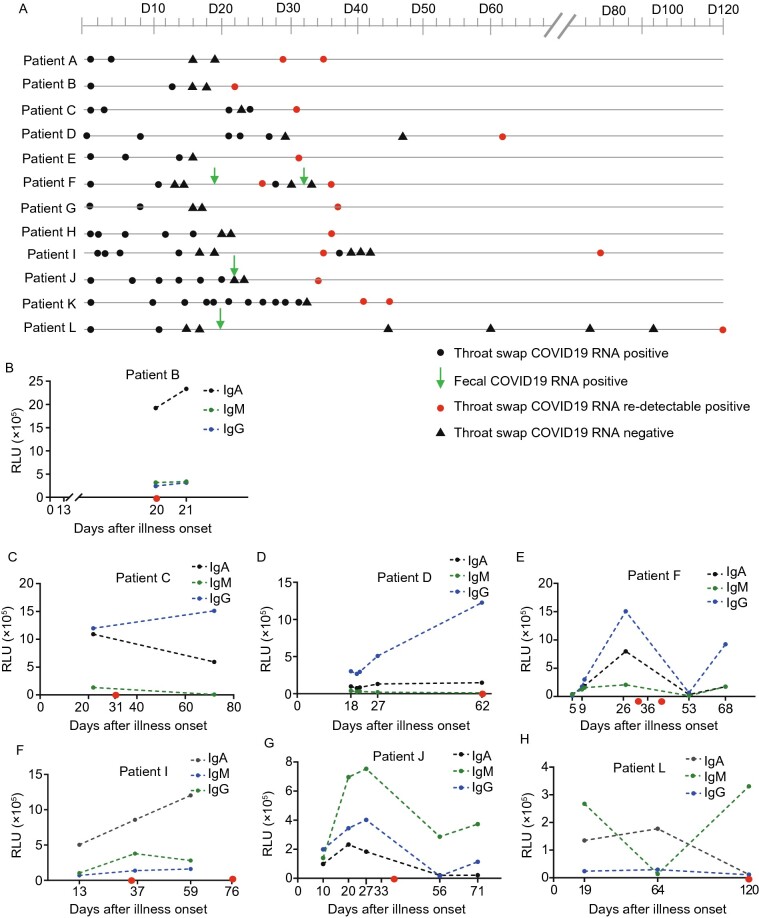
**Longitudinal changes of COVID-19 specific immunoglobulins in patients during RP**. (A) Throat swaps and fecal samples from COVID-19 RP patients were collected for RT-qPCR test. Days was count from the first day of confirmed SARS-CoV-2 RNA positive. The Black dot and triangle represents the time points of throat swap SARS-CoV-2 RNA positive and negative respectively. Red dot represents the time points of throat swap re-detectable SARS-CoV-2 RNA positive, and the green arrow represents the time points of fecal SARS-CoV-2 RNA positive. (B–H) Longitudinal changes of SARS-CoV-2 S protein-specific IgM, IgG and IgA in the serum of RP patients were quantified by ELISA. The red dots indicate time of RP

Fecal samples were collected at the time of discharge from five of the 12 patients who were subsequently RP, and 60% (three patients) were tested positive for SARS-CoV-2 RNA (Table S2). This rate was higher than the 11.3% (6 out of 53) SARS-CoV-2 RNA-positive rate for fecal samples from non-RP patients in Hefei (*P* = 0.026, chi-squared contingency table tests), which supported a potential causal relationship between intestinal infection and RP. Applying both untargeted and targeted metagenomics approaches, three near full-length SARS-CoV-2 sequences and one partial genome sequence were recovered from four fecal samples (Fig. S1), indicating active viral infection in the gastrointestinal tract of these patients.

Patient F experienced a fever and whole-body aches from February 6. Pneumonia was confirmed by CT scan on February 7, and the patient was confirmed to have COVID-19 by SARS-CoV-2 RNA test on February 9. The patient was transferred to ICU of the First Affiliated Hospital of USTC as a severe case on February 17. Patient F had a mild respiratory distress and shortness of breath, as well as cardiac insufficiency. He was given glucocorticoid, immunoglobin, antibiotics, antiviral agents, anticoagulant, and probiotics, and was supplied with oxygen. Symptom distress and cardiac function significantly improved during the patient's stay in ICU, and he was transferred out of ICU on February 22. SARS-CoV-2 RNA tests on throat swab samples from the patient were negative on February 21, and he was discharged from the hospital on February 24. A fecal sample collected from the patient at discharge tested positive for SARS-CoV-2 RNA. However, the discharge policy in Hefei according to the national instructions did not include fecal detection of SARS-CoV-2 RNA. After discharge, the patient remained in quarantine and was tested for SARS-CoV-2 RNA every three days. A throat swab sample was positive for SARS-CoV-2 RNA on March 5 and the patient was readmitted to hospital (Fig. 1A). The patient remained asymptomatic during his stay in hospital, and was discharged on March 12, although his fecal samples still tested positive for SARS-CoV-2 RNA. Throat swab samples from the patient were positive again for SARS-CoV-2 RNA on March 15 (Fig. 1A), possibly because of active SARS-CoV-2 infection in the intestine.

Serum samples were collected from six patients who were RP, and two showed an IgA-dominant signature of immunoglobulin production (Fig. 1B and 1F). This is indicative of a possible intestinal infection. Moreover, continuous serum samples (at least three time points) were collected from four out of 12 patients who were RP, and all of the samples showed an increase in SARS-Cov-2 specific antibody(s) production during RP (Fig. 1B–H). Notably, SARS-CoV-2 specific antibody titers (including IgM, IgG and IgA) for Patient F peaked at time of the RP (Fig. 1E), which is indicative of re-infection. Mucosal infection normally induces a high level of IgA, and re-infection results in an increase or boosting of humoral immune responses such as immunoglobin production. Thus the samples from the patients who were RP was analyzed for IgA production. SARS-CoV-2 specific IgA production was slightly increased in the serum of patients who were RP than in the serum of NRP controls (Fig. 2A), indicative of higher possibility of intestinal infection in RP patients, as most IgA is produced by gut mucosal production in mammals (Papista et al. 2011).

**Figure 2:**
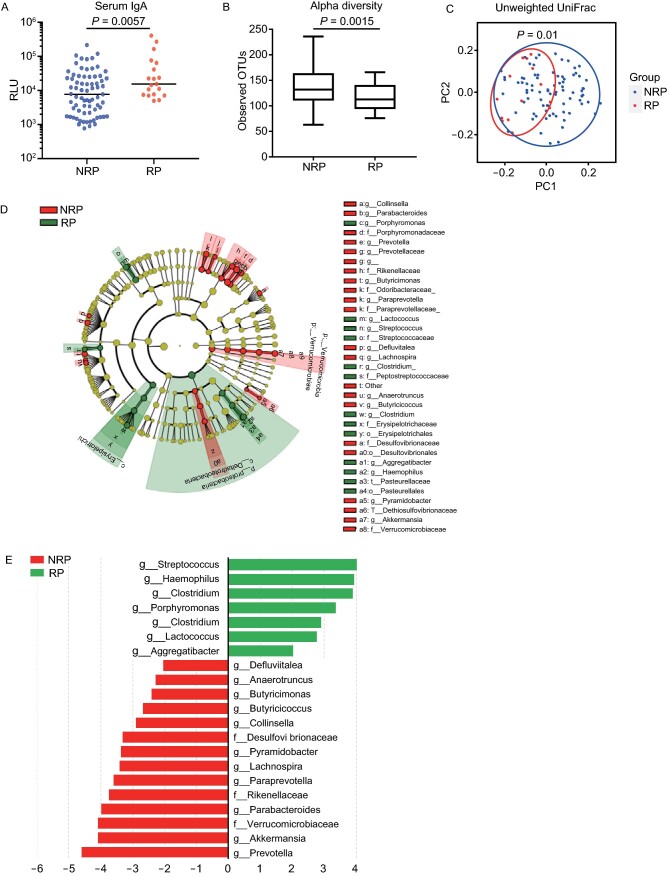
**Alternation of gut microbiota in RP patients**. (A) SARS-CoV-2 S protein-specific IgA in Serum samples of RP and NRP patients were measured by ELISA (mean ± s.e.m., Student's *t*-test). Fecal DNA from RP and NRP patients were analyzed by 16s sequencing. (B) Alpha diversity was calculated by observed OTU number in RP or NRP patients (mean ± s.e.m., Student's *t*-test). PCA plot generated from (C) unweighted UniFrac distance matrix displayed the distinct clustering pattern of intestinal bacteria community in RP patients and NRP controls. (D and E) Composition differences were determined by linear discriminant analysis using LEfSe, and differential features were showed on taxonomic trees (D), or ranked by effect size (E)

Enteric viral infection frequently results in intestinal dysbiosis (Ma et al., 2019), the bacterial 16S ribosome of the fecal samples were sequenced to further examine the association between possible intestinal infection with clinical outcomes of RP patients. There was decreased diversity in commensal bacteria in the intestine of patients who were RP compared with that from NRP group (Fig. 2B). Further, there was a distinct pattern of difference in the microbiota composition between patients who were RP and NRP group (Fig. 2C). Patients who were RP showed an increase in the bacterial genera *Streptococcus, Clostridium, haemophilus, Proteobacteria*, and a decrease in *Prevotella, Akkermansia, Paraprevotella*, and *Lachnospira* (Fig. 2D and 2E). Notably, none of 8 patients with *Prevotella* rich microbiota were RP. It would be interesting to verify whether patients with *Prevotella* enterotype were less likely became RP in larger cohorts.

Several studies have been performed to investigate active SARS-CoV-2 replication in the gastrointestinal tract (Xiao et al., 2020; Lamers et al., 2020). The angiotensin-converting enzyme 2 (ACE2), to which SARS-CoV-2 binds, is highly expressed in the intestine (Liang et al., 2019). Human intestinal organoids, which mimic the specific cell type and spatial structure of the intestine, have been shown to be susceptible to SARS-CoV2 infection (Lamers et al., 2020). Enteroscopy and biopsy on a patient with COVID-19 showed numerous infiltrating plasma cells and lymphocytes, with interstitial edema in the lamina propria of the stomach, duodenum, and rectum (Xiao et al., 2020). Moreover, viral RNA in fecal samples may persist for more than 30 days (Wu et al., 2020). These results indicate that, in addition to the lungs, SARS-CoV-2 may also reside in the gastrointestinal tract. Consistent with this idea, we successfully recovered three almost full-length, and one partial viral genome sequences from fecal samples. We also observed alternation of the intestinal microbiota in fecal samples of patients who were RP, which may further contribute to gastroenteritis-like symptoms (Wang et al., 2014). Further, our study revealed that IgA, which are mainly produced in the intestine, were induced during infection with SARS-CoV-2.

There are some limitations to our study. Firstly, the lack of a P3 facility meant that we were unable to test whether live virus could be isolated from fecal samples of patients who were RP after the first discharge from hospital. If this could be ascertained, then it would be direct evidence to support the “intestinal reservoir” theory. The second limitation of the study is the relatively small sample size. In the study, 12/12 (100%) patients who were RP had gastrointestinal symptoms on their first admission to hospital; fecal samples of 3/5 (60%) patients who were RP were positive for SARS-CoV-2 RNA; and 5/6 (83.3%) patients who were RP showed a boost in immunoglobulin responses during RP. Notably, previous studies reported that RP usually appears in patients with mild to moderate COVID-19 symptoms at the first admission (An et al., 2020; Lan et al., 2020). In contrary to this observation, among the 12 patients who were RP in this study, there were only five mild/moderate cases of COVID-19, but five severe cases and two critical cases. Further studies with larger cohorts might be useful to determine the correlation between intestinal infection with SARS-CoV-2 and RP.

Understanding the mechanism(s) underlying RP may provide useful information for establishing standards for discharge and follow-up of patients recovering from COVID-19. Notably, one patient in our study (Patient L) re-tested positive for SARS-CoV-2 RNA three months after her discharge from hospital, implying that this virus may last long in the patient. Thus, our results strongly suggest that negative fecal detection (at least rectal swab detection) should be included as one of the criteria for hospital discharge of patients recovering from COVID-19. Shanghai is the only city in China that currently includes a fecal SARS-CoV-2 RNA test as part of the hospital discharge policy, and this city has a much lower RP rate than other cities (<1% in Shanghai vs. overall 5%–15% in China, NHC, http://www.nhc.gov.cn/xcs/s3574/202005/dd9e76dabf154c1cafc8dba40c57eac9.shtml). In summary, our results suggest that the intestine might be the “reservoir” of SARS-CoV-2 and one of the direct causes of RP. The findings of this study provide useful information on the pathogenesis of SARS-CoV-2 infection and highlight the need for stricter hospital discharge criteria to include being fecal-detection-negative for SARS-CoV-2 RNA.

## Electronic supplementary material

The online version of this article (https://doi.org/10.1007/s13238-020-00778-8) contains supplementary material, which is available to authorized users.

## Supplementary Material

13238_2020_778_MOESM1_ESM
