# Supplementary material for: Re-detectable positive SARS-CoV-2 RNA tests in patients who recovered from COVID-19 with intestinal infection
Source: Protein Cell. 2020 Sep 26;12(3):230–5. doi: 10.1007/s13238-020-00778-8 (PMC7518948; doi:10.1007/s13238-020-00778-8)
Supplement: 13238_2020_778_MOESM1_ESM [file 13238_2020_778_moesm1_esm.pdf]

METHODS

Cases

A total of 173 patients who had COVID-19 and were discharged from hospital in Hefei, China between January 21 and March 8, 2020, were enrolled in this study for analysis of their clinical parameters, and were followed-up for at least one month. Patient history, physical findings, and hematological, biochemical, and microbiological investigation results were recorded and analyzed. All laboratory procedures for clinical samples have been previously reported(Xu et al., 2020; Zuo et al., 2020). Briefly, throat swabs and fecal samples were collected into viral transfer media. Serum were separated from clotted blood bottles. This study was approved by the Institutional Review Board of the University of Science and Technology of China. Patients or the public were not but may involved later in the design, or conduct, or reporting, or dissemination plans of our research

In-house one-step real-time RT-PCR assay

Throat swabs and fecal samples were pretreated with heat incubation. Approximately 5 g of fecal sample was suspended in 5 ml normal saline solution, homogenized thoroughly, and centrifuged for 5 min at 4,000 rpm. The fecal extracts were prepared for viral RNA extraction and IL-18 measurement. For RNA extraction, 230 μl throat swab or fecal extract were processed using a Viral DNA/RNA extraction kit (Magnetic beads) in a SSNP-3000A Nucleic Acid Extraction System (Jiangsu Shuo Shi Biotechnology Co., Ltd, China). Ten microliters of the resulting RNA were used for real-time RT-PCR targeting the ORF1ab gene of SARS-CoV-2, using an RT-PCR kit from BGI genomics (Shenzhen, China) according to the manufacturer's instructions. Cycling conditions of 50°C for 20 min, 95°C for 10 min, followed by 40 cycles of

95°C for 15 s and 60°C for 30 s, were performed with the StepOnePlus Real-Time PCR System (Applied Biosystems, USA). Samples were considered positive when the Ct values of the target gene were ≤38 cycles. The copy numbers of viral nucleic acid in each sample was calculated according to the qRT-PCR results of orf1b as well as the standard curve formula $y=-3.3308x+39.598$ $R^2=0.9937$(Chu et al., 2020).

Enzyme-linked immunosorbent assay (ELISA) analysis of IL-18

Fecal extracts were prepared as described above and IL-18 levels were measured using an IL-18 ELISA Kit (Sino Biological) according to the instructions of the vendor.

16S rRNA gene sequencing of fecal microbiota

Fecal samples were stored at -80°C until 16S rRNA gene analysis. DNA was extracted from fecal pellets with a QIAamp stool DNA Mini kit (QIAGEN) according to the manufacturer's instructions. 16S rRNA gene amplicons were generated using the primer pair 515F/806R as recommended by Earth Microbiome Project(Thompson et al., 2017). PCR products were quantified, pooled, cleaned using the PCR Cleanup kit (QIAGEN), and subsequently sequenced on Illumina MiSeq(2X250bp). Custom primers were added to the Illumina MiSeq kit resulting in a 253-bp fragment, and following paired-end joining, sequencing was achieved to a depth of 16,301± 14,760 reads (mean ± SD). Microbial diversity was analysed by usearch(v8.1)(Edgar, 2010) and Qiime(v1.9.1)(Caporaso et al., 2010). The linear discriminant analysis effect size Galaxy module

(http://huttenhower.sph.harvard.edu/galaxy/) was used for additional statistical analyses(Segata et al., 2011).

Read 1: TATGGTAATTGTGTGCCAGCMGCCGCGGTAA

Read 2: AGTCAGTCAGCCGGACTACHVGGGTWTCTAAT

Index sequence primer: ATTAGAWACCCBDGTAGTCCGGCTGACTGACTATTAGAA

Measurement of serum immunoglobins

SARS-CoV-2-specific IgA, IgM, and IgG detection kits using chemiluminescence were developed by Kangrun Biotech (Guangzhou, China). The receptor-binding domain of the spike protein was coated onto magnetic particles to catch SARS-CoV-2-specific IgA, IgM and IgG in patient samples. A second antibody, which recognizes IgA, IgM, or IgG, conjugated with acridinium (which can react with substrates to generate strong chemiluminescence) was used for detection of IgA, IgM, and IgG, respectively. The detected chemiluminescence signal over the background signal was calculated as relative light units (RLU). The detection kits have been validated using a large cohort of serum samples, and show high sensitivities and specificities(Ma et al., 2020) . Prior to testing, a denaturant solution was added to each serum sample to a final concentration of 1% TNBP, 1% Triton X-100. After mixing by inversion, the samples were incubated at 30°C for 4 h to completely denature any viruses.

Viral amplification and sequencing

For generation of viral genomes, untargeted and targeted metagenomics approaches were used. An untargeted metagenomic sequencing library was prepared with a Trio RNA-Seq kit (NuGEN Technologies, USA) according to a previous study(Chen et al., 2020), and was

sequenced on Illumina HiSeq sequencing platforms. For the targeted approach, version 1 of the ARTIC COVID-19 multiplex PCR primers (https://artic.network/ncov-2019) were used, followed by next-generation sequencing library preparation (NEBNext Ultra DNA Library Prep Kit for Illumina) and sequencing on Illumina NovaSeq sequencing platforms. Raw reads were processed with fastp v0.20.0 to remove Illumina adaptors and filter low-quality reads (q30 threshold and read length >15 nt)(Chen et al., 2018). Mapping of clean reads was performed against the SARS-CoV-2 reference strain Wuhan-Hu-1 (GenBank accession number MN908947.3) using Bowtie2 v2.3.2 (Langmead and Salzberg, 2012). Consensus sequences were generated using samtools 1.7(Li et al., 2009) .

Statistical analysis

The sample size selected for experiments in this study was estimated based on previous similar experiments. For all bar graphs, data were expressed as mean ± SEM. A standard two-tailed unpaired Student's *t*-test or paired Student's *t*-test was performed using GraphPad Prism 6. To compare two non-parametric datasets, a Mann–Whitney U-test was used. p values ≤0.05 were considered to indicate significance. Sample sizes (biological replicates), specific statistical tests, and the main effects of the statistical analyses for each experiment are detailed in each figure legend.

Reference

Caporaso, J.G., Kuczynski, J., Stombaugh, J., Bittinger, K., Bushman, F.D., Costello, E.K., Fierer, N., Pena, A.G., Goodrich, J.K., Gordon, J.I.*, et al.* (2010). QIIME allows analysis of high-throughput community sequencing data. Nature methods *7*, 335-336.

Chen, L., Liu, W., Zhang, Q., Xu, K., Ye, G., Wu, W., Sun, Z., Liu, F., Wu, K., Zhong, B.*, et al.* (2020). RNA based mNGS approach identifies a novel human coronavirus from two individual pneumonia cases in 2019 Wuhan outbreak. Emerging microbes & infections *9*, 313-319.

Chen, S., Zhou, Y., Chen, Y., and Gu, J. (2018). fastp: an ultra-fast all-in-one FASTQ preprocessor. Bioinformatics *34*, i884-i890.
Chu, D.K.W., Pan, Y., Cheng, S.M.S., Hui, K.P.Y., Krishnan, P., Liu, Y., Ng, D.Y.M., Wan, C.K.C., Yang, P., and Wang, Q.J.C.C. (2020). Molecular Diagnosis of a Novel Coronavirus (2019-nCoV) Causing an Outbreak of Pneumonia.
Edgar, R.C. (2010). Search and clustering orders of magnitude faster than BLAST. Bioinformatics *26*, 2460-2461.
Langmead, B., and Salzberg, S.L. (2012). Fast gapped-read alignment with Bowtie 2. Nature methods *9*, 357-359.
Li, H., Handsaker, B., Wysoker, A., Fennell, T., Ruan, J., Homer, N., Marth, G., Abecasis, G., and Durbin, R. (2009). The Sequence Alignment/Map format and SAMtools. Bioinformatics (Oxford, England) *25*, 2078-2079.
Ma, H., Zeng, W., He, H., Zhao, D., Yang, Y., Jiang, D., Zhou, P., Qi, Y., He, W., Zhao, C.*, et al.* (2020). COVID-19 diagnosis and study of serum SARS-CoV-2 specific IgA, IgM and IgG by a quantitative and sensitive immunoassay.
Segata, N., Izard, J., Waldron, L., Gevers, D., Miropolsky, L., Garrett, W.S., and Huttenhower, C. (2011). Metagenomic biomarker discovery and explanation. Genome Biol *12*, R60.
Thompson, L.R., Sanders, J.G., McDonald, D., Amir, A., Ladau, J., Locey, K.J., Prill, R.J., Tripathi, A., Gibbons, S.M., Ackermann, G.*, et al.* (2017). A communal catalogue reveals Earth's multiscale microbial diversity. Nature *551*, 457-463.
Xu, X., Han, M., Li, T., Sun, W., Wang, D., Fu, B., Zhou, Y., Zheng, X., Yang, Y., Li, X.*, et al.* (2020). Effective treatment of severe COVID-19 patients with tocilizumab. Proceedings of the National Academy of Sciences of the United States of America *117*, 10970-10975.
Zuo, T., Zhang, F., Lui, G.C.Y., Yeoh, Y.K., Li, A.Y.L., Zhan, H., Wan, Y., Chung, A., Cheung, C.P., Chen, N.*, et al.* (2020). Alterations in Gut Microbiota of Patients With COVID-19 During Time of Hospitalization. Gastroenterology.

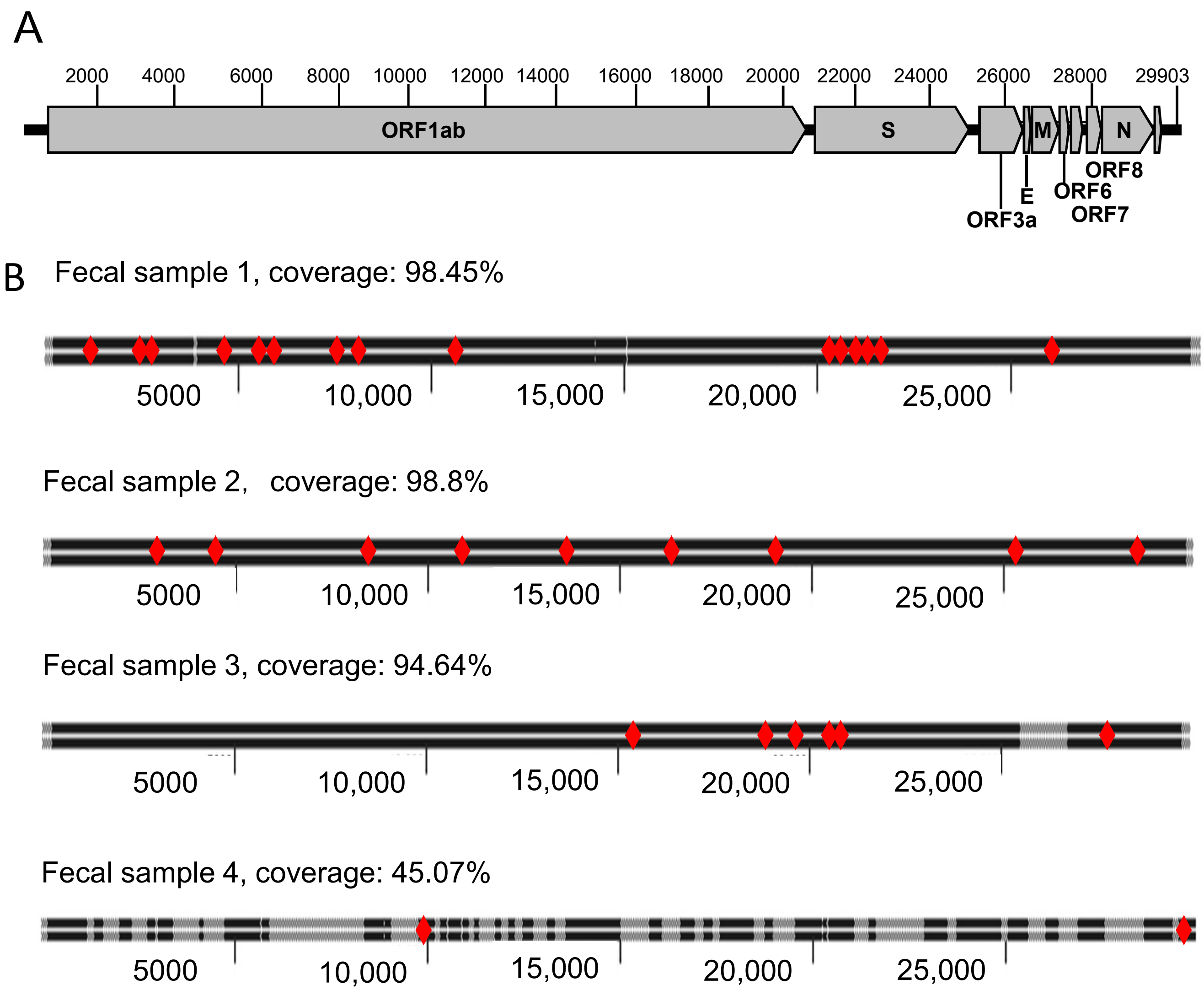

Figure S1. The genome coverage map for SARS-CoV-2 sequences in fecal samples from 4 COVID-19 patients. (a) Genomic structure of COVID-19. (b) 3 near full-length SARS-CoV-2 and 1 partially genome sequence was recovered from 4 fecal samples. Black color represent sequencing covered region and red dot indicate SNPs according to reference genome Wuhan-Hu-1 (GenBank accession number MN908947.3)

Table S1. Clinical and pathological characteristics of the COVID-19 RP patients

Patients	A	B	C	D	E	F	G	H	I	J	K	L
**Characteristics**												
Age，year	36	65	62	57	53	68	47	33	35	45	53	24
Gender	Male	Female	Male	Male	Male	Male	Male	Female	Male	Female	Female	Female
Occupation	Office worker	Farmer	Self-employed	Farmer	Farmer	Retired	Doctor	Retired	Freelancer	Retired	Retired	Factory worker
Current smoking status	No	No	No	No	No	No	No	No	No	No	No	No
Current alcohol drinking	No	No	No	No	No	No	Yes	No	Yes	No	No	No
Comorbidities	No	Diabetes	Diabetes, Chronic hepatitis B	Diabetes	No	Hypertension, Diabetes, Coronary heart disease	Hypertension	No	Chronic hepatitis B	No	Hypothyroidisr	No
**Respiratory and systemic symptoms**												
Fever	+	+	+	+	-	+	+	+	+	+	+	-
Cough	+	+	+	+	+	-	+	+	+	-	-	+
Sputum	-	+	+	-	-	-	+	+	-	-	-	+
Chest congestion	+	+	+	+	+	+	+	-	+	-	-	-
Shortness of breath	-	+	+	+	-	-	+	-	-	-	-	-
Fatigue	+	-	+	-	+	-	-	-	+	-	-	-
Dizziness	-	-	-	-	+	-	-	-	-	-	+	-
Headache	-	-	-	+	-	-	-	-	-	-	-	-
Myalgia	-	-	-	-	+	+	-	-	-	-	-	-
Anorexia	+	-	+	-	-	-	-	+	-	-	-	-
Temperature (°C)	37.7	37.5	39.0	37.6	37.3	38.8	38.5	37.4	38.4	38.0	37.3	36.2
**Treatments**												
Corticosteroids	+	+	+	+	+	+	+	-	+	-	-	-
Immunoglobin	+	+	+	+	+	+	+	-	-	-	-	-
Oxygen therapy	+	+ (High-flow)	+ (High-flow)	+ (High-flow)	+	+	+ (High-flow)	-	-	-	-	-
Mechanical ventilation	-	-	-	-	-	-	-	-	-	-	-	-
Antiviral treatment	+	+	+	+	+	+	+	+	+	+	+	+
Antibiotics	+	+	+	+	+	-	+	-	+	+	-	+
Traditional Chinese medicine	+	-	-	+	-	+	+	+	+	+	+	+
Probiotics	+	-	+	+	+	+	+	-	+	-	-	-
Anticoagulation	-	-	-	+	+	+	-	-	-	-	-	-
Tocilizumab treatment	-	+	-	-	-	+	+	-	-	-	-	-
ECMO	-	-	-	-	-	-	-	-	-	-	-	-
ICU Adimission	No	Yes	No	Yes	No	Yes	Yes	No	No	No	No	No
Complications	Influenza B virus coinfection, Mycoplasma coinfection	Influenza A virus coinfection, Mycoplasma coinfection, Respiratory failure, Shock, Heart	No	No	Acute cardiac injury	Cardiac insufficiency	Respiratory failure	No	No	No	No	No
Outcomes	Discharged	Discharged	Discharged	Discharged	Discharged	Discharged	Discharged	Discharged	Discharged	Discharged	Discharged	Discharged
Days from the onset of illness to hospital admission	1	2	2	3	1	1	1	1	5	1	7	3
Days from the onset of illness to SARS-CoV-2 RNA positive	2	7	3	8	6	3	8	5	8	1	7	3
Days from the onset of illness to SARS-CoV-2 RNA negative	15	21	24	27	17	15	16	19	21	19	28	10
Days from hospital admission to discharge	21	22	25	29	21	22	24	26	20	24	30	12
Days from discharge to re-detectable SARS-CoV-2 RNA positive	7	13	5	2	13	5	13	13	15	9	8	102
**Laboratory data**												
White blood cell count, × 10^9/L (normal range 3.5-9.5)	5.53	10.35	6.57	6.47	7.54	3.52	5.93	4.04	7.73	6.87	5.33	5.35
Neutrophil count, × 10^9/L (normal range 1.8-6.3)	3.87	9.53	5.63	4.75	5.84	2.27	5.11	1.60	5.09	5.77	3.88	2.31
Lymphocyte count, × 10^9/L (normal range 1.1-3.2)	0.89	0.42	0.57	1.19	1.01	1.03	0.52	2.07	1.83	0.54	1.05	2.53
Haemoglobin, g/L (normal range 115-150)	150	110	138	154	126	143	140	114	155	116	111	125
Platelet count, × 10^9/L (normal range 125-350)	147	106	205	108	259	88	183	164	143	195	144	170
Albumin，g/L (normal range 40-55)	39.8	33.7	30.1	44.2	31.0	37.4	32.9	45.3	52.0	46.8	43.1	47.2
Alanine aminotransferase, IU/L (normal range 7-40)	24	27	32	50	43	22	16	10	171	13	20	10
Aspartate aminotransferase, IU/L (normal range 13-40)	27	17	23	42	21	36	19	20	60	22	25	18
Bilirubin, µmol/L (normal range 3.4-21.0)	20.0	22.2	30.3	12.4	12.4	25.1	11.8	14.7	14.2	16.5	66.8	15.1
Urea, mmol/L (normal range 2.6-7.5)	5.88	7.83	7.95	2.98	6.66	4.94	5.99	3.28	3.56	3.08	3.87	4.59
Creatinine, µmol/L (normal range 41-81)	57	67	75	74	69	73	77	60	79	51	73	60
Cystatin C, mg/L (normal range 0.59-1.03)	1.49	2.01	1.40	1.16	1.39	1.54	0.97	0.75	0.96	0.45	0.73	0.48
Lactate dehydrogenase, U/L (normal range 120-250)	270	280	273	253	215	480	311	168	202	175	168	246
Creatine kinase, IU/L (normal range 22-269)	158.3	42.1	87.0	34.8	33.8	177.2	214.9	62.7	194.7	152.8	54.2	61.9
High-sensitive cardiac troponin I, µg/mL (normal range 0-0.3)	0.11	0.40	0.11	1.32	1.00	0.35	0.09	0.12	0.29	0.79	0.07	0.05
Pro-brain natriuretic peptide, pg/mL (normal range 0-150)	100.00	2544.00	98.00	48.21	NA	1907.00	89.00	NA	NA	NA	196.00	NA
Prothrombin time, s (normal range 9.5-14.5)	13.6	15.5	17.6	14.0	14.5	16.3	14.3	13.2	14.7	14.1	14.4	14.0
Activated partial thromboplastin time, s (normal range 20-40)	32.3	38.7	32.3	40.6	27.5	39.2	29.7	35.4	44.0	36.3	39.9	38.4
D-dimer, µg/mL (normal range 0-1. 1)	0.24	0.36	1.27	0.12	0.70	0.25	3.93	0.10	0.15	0.22	0.05	0.19
IL-6, pg/mL (normal range ＜7.0)	6.41	6.87	7.06	6.52	7.25	56.23	7.78	5.21	5.69	1.70	5.10	1.55
Procalcitonin, ng/mL (normal range 0-0.5)	0.15	＜0.10	0.29	0.21	0.11	0.16	0.18	0.13	0.18	＜0.10	＜0.10	0.10
C-reactive protein, mg/L (normal range 0-8.0)	36.4	29.7	109.9	19.4	23.5	57.8	101.6	3.8	21.4	12.6	0.6	0.5

Table S2. Evidence of Gastrointestinal infection of SARS-CoV-2

Patients	Confirmed on	RP on	Gastro-intestinal Symptome (100%) literatures 2%-39%	Fecal Detection on Discharge (50%) Overall 15%	High IgA level (83%)	IL-18 level	Increases of Immunoglobins during RP (83%)	symptoms type
A	Jan 28;	Feb 25; Mar 2	Diarrhea , Anorexia, Nausea	--	Yes	--	Yes	Severe
B	Jan 30;	Feb 25;	Anorexia	--	Yes	--	Yes	Critical
C	Jan 29;	Feb 28;	Anorexia , Nausea , Vomit	--	No	low	No	Severe
D	Jan 28;	Mar 3;	Vomit	No	No	high	Yes	Severe
E	Jan 29;	Mar 4;	Diarhea , Anorexia ,Vomit , Epigastric discomfort	--	--	--	--	Severe
F	Feb 9;	Mar 5; Mar 15	Diarrhea, Vomit, Epigastric discomfort	Yes	Yes	low	Yes	Critical
G	Feb 4;	Mar 5;	Diarhea ,Anorexia (Onset)	--	--	--	--	Severe
H	Jan 30;	Mar 6;	Diarhea , Anorexia (Onset)	--	--	--	--	General
I	Feb 1;	Mar 7;	Diarrhea	No	Yes	low	Yes	General
J	Feb 6;	Mar 10;	Diarhea ,Vomit	Yes	Yes	high	Yes	General
K	Feb 2;	Mar 13; Mar 19	Stomach discomfort	--	No	high	No	General
L	Jan 28;	May 28;	Diarrhea	Yes	--	--	--	General
